# Sex-based differences in clinical and radiological presentation of patients with degenerative lumbar scoliosis: a cross-sectional study

**DOI:** 10.1186/s13018-023-04357-5

**Published:** 2023-12-21

**Authors:** Xiao Liang, Puxin Yang, Hongru Yuan, Yachong Huo, Dalong Yang, Hui Wang, Wenyuan Ding

**Affiliations:** Department of Spinal Surgery, Hebei Medical University Third Hospital, Shijiazhuang, China

**Keywords:** Spinal deformity, Degenerative lumbar scoliosis, Cross-sectional study, Sex-gender differences, Curve progression

## Abstract

**Background:**

To evaluate the sex-based differences in clinical and radiological presentation of patients with degenerative lumbar scoliosis (DLS) and to explore whether the difference is helpful in investigating the etiology and progression of DLS.

**Method:**

A retrospective review of 199 patients (41 males, 158 females) with DLS was included. Patient demographics including age, gender, bone mineral density were collected. Back and leg pain was assessed by visual analog scale, and general physical condition was assessed by Oswestry Disability Index. Cobb’s angle was measured, and direction of scoliosis, position of the superior, inferior and apex vertebrae, number of vertebrae included in the scoliosis, rotation of apex vertebrae (Nash-Mo index), translation of apex vertebrae were recorded. Sagittal longitudinal axis, thoracolumbar kyphosis, lumbar lordosis (LL), pelvic incidence angle (PI), sacral slope, apex of lumbar lordosis and coronal balance distance were measured by whole spine lateral radiographs, and type of coronal imbalance was evaluated in all patients. Fat infiltration rate (FIR) of the paraspinal muscles at the vertebral apex was measured by MRI.

**Result:**

Compared to female patients, male patients showed more back and leg pain on clinical presentation and smaller Cobb angle, less parietal rotation, larger LL, smaller PI-LL and lower paravertebral muscle FIR on radiologic features.

**Conclusion:**

Gender differences do exist in DLS patients with regard to clinical and radiological presentation, low back pain was more pronounced in male patients, and scoliosis was more severe in female patients based on this cross-sectional study.

## Background

Male and female patients always present differences in clinical and radiological presentations even if they are diagnosed to the same orthopedic disorder, which may be helpful in understanding the etiology and pathological process of the disease based on the sex difference. Dror et al. [1] proved that male and female patients differ in their hip structure, biomechanics and operative findings of symptomatic labral tears, males are more likely to report an acute injury and females are more likely to be evaluated with increased range of motion. Nadine [2] demonstrated that males and females may be exposed to different loading patterns during prolonged sitting and may experience different pain generating pathways, and gender-dependent treatment modalities and/or coaching should be implemented when considering methods of reducing the risk of injury or aggravation of an existing injury.

Degenerative lumbar scoliosis (DLS) is a three-dimensional disorder that mostly affects the skeletal mature patients [3, 4]. Xu et al. [5] demonstrated that the prevalence of DLS in Chinese Han population aged older than 40 years was approximately 13.3%, which had a significant correlation with gender. The comparison of prevalence between genders revealed that female subjects had an obviously higher incidence of lumbar scoliosis than male subjects. This prospective study of 2,395 individuals also confirmed that bone mineral density (BMD) is an independent influence on the development of DLS. Differences in BMD with age between male and female subjects, especially the sharp decline in BMD in postmenopausal women, partly explain the different incidence of DLS in men and women. However, no previous study specifically focus on the sex-based differences in patients with DLS. The purpose of the current study is therefore to evaluate the sex-based differences in clinical and radiological presentation of patients with DLS and to explore whether the difference is helpful in investigating the etiology and progression of DLS.

## Methods

### Subjects

This study was approved by the Institutional Review Board of our hospital before data collection and analysis.(ID: 2022-094-1) Inclusion criteria were: (1) DLS patients with age older than 45 years, (2) full-spine postero-anterior (P/A) and lateral X-ray, (3) lumbar CT was available for Hounsfield unit (HU) values measurement, (4) lumbar MRI was available for evaluating paraspinal muscle degeneration. Exclusion criteria were: (1) previous surgery for degenerative lumbar disease, (2) patients those with olisthesis, lumbarization, sacralization, rheumatic/neurologic/endocrine diseases and spinal infections, (3) the anatomical identification was difficult to recognize for radiological measurement. By retrieving the medical records from January 2011 to December 2020 in our hospital, 199 patients who met both the inclusion and exclusion criteria were retrospectively reviewed and enrolled in the current study.

### Data collection and assessment

Patient demographic data including age, gender, BMD, back pain and leg pain evaluated by visual analog scale (VAS), overall physical condition evaluated by Oswestry Disability Index (ODI) [6] were collected from the medical records.

**Fig. 1:**
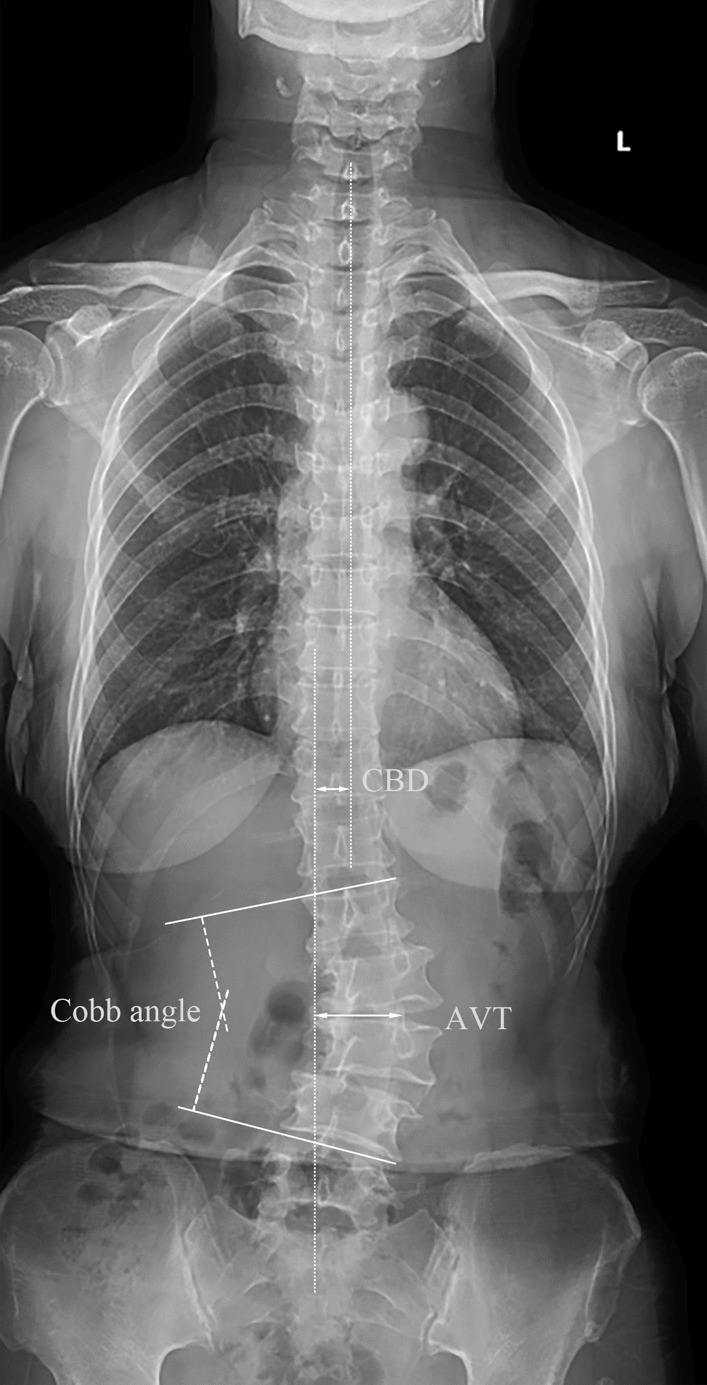
Schematic diagram of coronal spine parameters measurement. The Cobb’s angle is measured between the most inclined vertebrae. Coronal balance distance (CBD) is the distance between the C7 plumb line and the central sacral vertical line (CSVL). Apical vertebral translation (AVT) is the horizontal distance from the CSVL to the apex of the vertebral body or the center of the lumbar disk

**Fig. 2:**
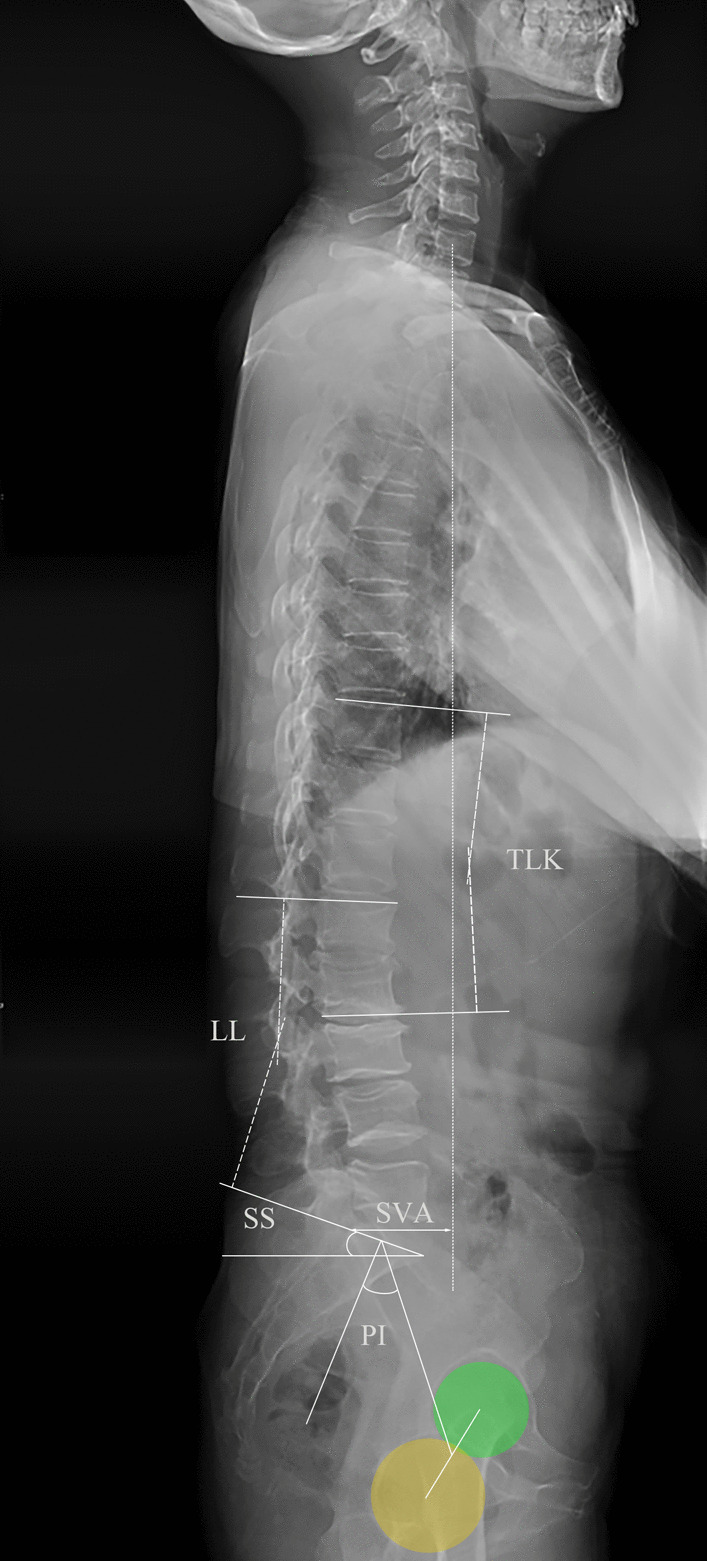
Schematic diagram of sagittal spine parameters measurement. Thoracolumbar kyphosis (TLK) is the angle between T10 and L2. Lumbar lordosis (LL) is the angle between L1 and S1. Pelvic incidence (PI) is the angle between the vertical line at the midpoint of the upper endplate of S1 and the midpoint of the line connecting the centers of the femoral heads. Sacral slope (SS) is the angle between the upper endplate of S1 and the horizontal line. Sagittal vertical axis (SVA) is the distance from the upper posterior corner of S1 to the C7 plumb line

Radiographic evaluation was performed based on the full-spine P/A and lateral X-ray. Cobb’s angle was measured between the most tilted vertebrae. Scoliosis orientation, location of upper end vertebrae, lower end vertebrae and apex vertebrae (AV), number of vertebrae included in the scoliosis, rotation of apex vertebrae (Nash-Moe index), apical vertebral translation (AVT) were recorded. Coronal balance distance (CBD) was the distance between C7 plumb line and central sacral vertical line (CSVL). (Fig. 1) Type of coronal imbalance was evaluated in all the patients: Type A, CBD < 3 cm; Type B, CBD > 3 cm and C7PL shifts to the concave side of the curve; and Type C, CBD > 3 cm and C7PL shifts to the convex side of the curve [7]. Sagittal vertical axis (SVA), thoracolumbar kyphosis (TLK), lumbar lordosis (LL), pelvic incidence (PI), sacral slope (SS) and apex of lumbar lordotic (aLL) were measured based on the full-spine lateral X-ray. (Fig. 2) For AV and aLL records, vertebrae from L1 to L5 were assigned numbers ranging from 1 to 5 to facilitate data analysis. If the apex is located within the disk between two vertebrae, a value of 0.5 is added to the number of the previous vertebra. For example, if the apex is located in the disk between L1 and L2, it is recorded as 1.5 [8].

**Fig. 3:**
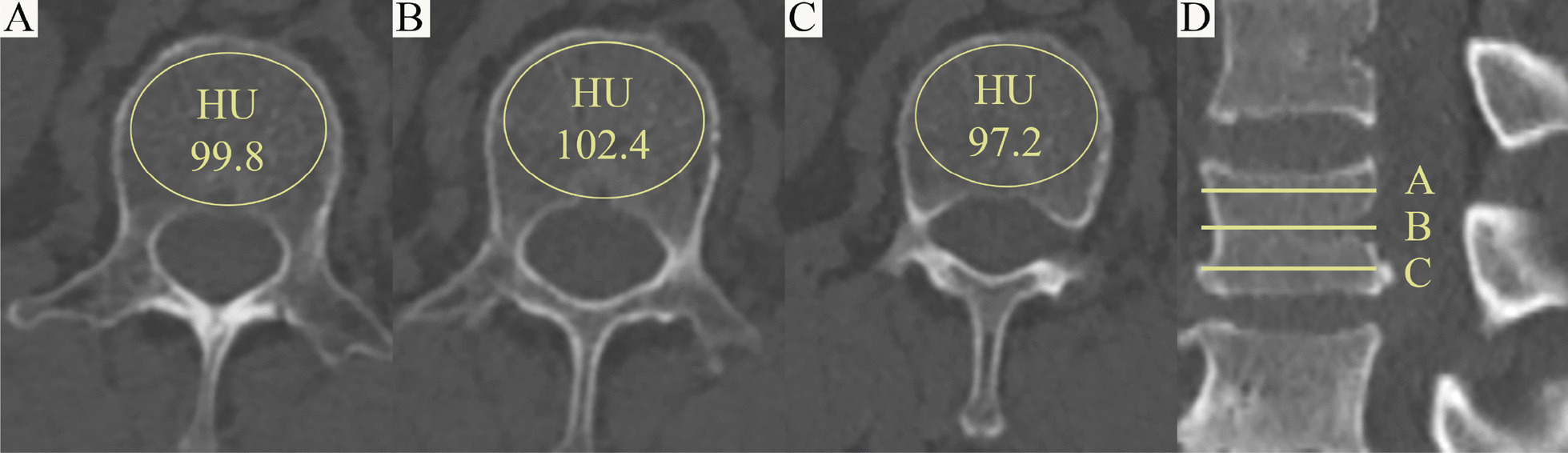
Schematic diagram of HU value measurement. HU values were measured in CT axial slice images of the patient’s L1 vertebrae (D) below the upper endplate (**A**), in the middle (**B**) and above the lower endplate (**C**), respectively, and averaged as the patient’s BMD. In each measurement, the largest possible elliptical region of interest was plotted, but the cortical margin was excluded to prevent volume averaging

**Fig. 4:**
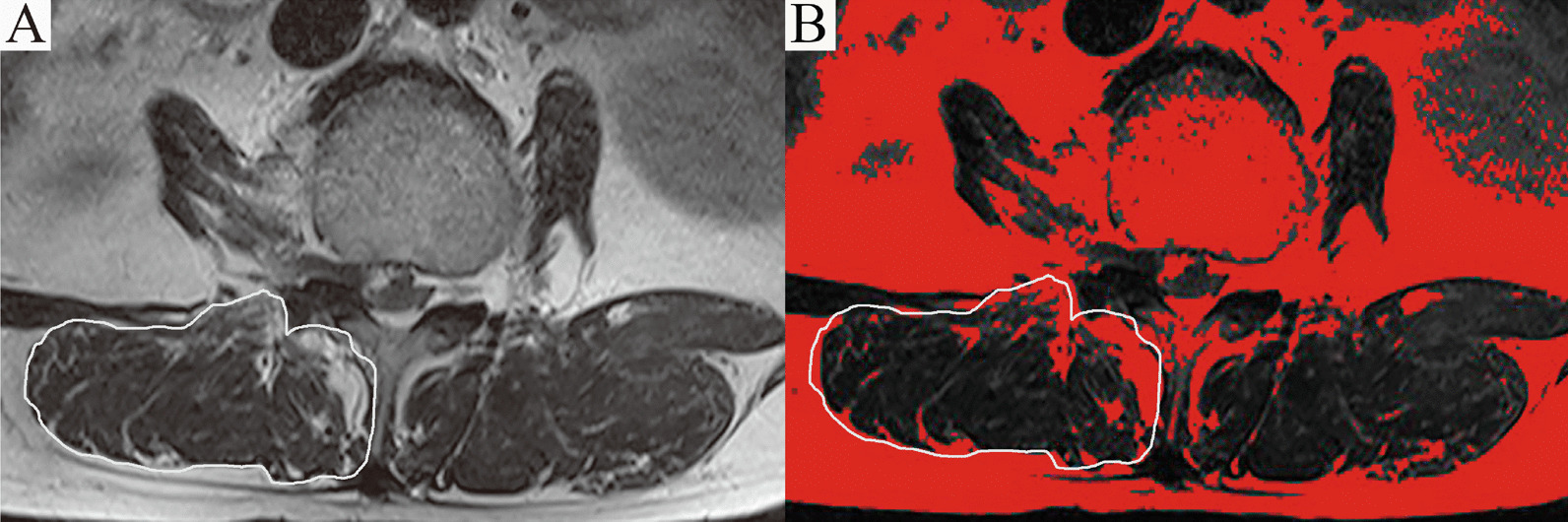
Schematic diagram of fatty infiltration rate (FIR) measurement. **A** Paraspinal muscles (multifidus and erector spinae) at the level of the apical vertebrae (or intervertebral disks) were circled on MRI. **B** A threshold technique was applied to color pixels of fat tissue within the muscles in red

The HU value of the patient’s L1 vertebrae represents the patient’s BMD. The HU values measurement within L1 vertebrae was obtained by using a protocol described by Schreiber [9]. (Fig. 3) Fatty infiltration rate (FIR) of paraspinal muscles (multifidus and erector spinae) at apical vertebrae was calculated by subtracting the muscle without the fat value from the total muscle value, and the images were adjusted with the image processing software (Image J, version 1.48, USA). (Fig. 4)

All radiographical parameters were measured by two independent observers (first author and second author) and were averaged to give a mean value for statistical analysis.

### Statistical analysis

Data were analyzed using Statistical Product and Service Solutions software (version 26; SPSS, Chicago, IL). Continuous variables satisfying a normal distribution were recorded as mean ± standard deviation, and categorical variables were expressed as number of cases. An independent t test was used to analyze the difference of continuous variables. An χ2 analysis and Fisher’s exact test were used to examine the differences among categorical variables. Spearman’s correlation analysis was used to test the correlation between the parameters. The statistical significance was set at *p* < 0.05.

## Results

### Characteristics of the subjects

Among the 199 patients included in the current study, 41 were males and 158 females, with mean age of 63.3±6.5 years. Coronal imbalance type A was detected in 145 patients, type B was detected in 28 patients, and type C was detected in 26 patients. 121 patients presented apex orientation toward left, and 78 patients presented apex orientation toward right. The mean Cobb angle was 25.5±11.1 degrees, and mean CBD was 22.8±18.7 mm.

### Comparison of clinical presentation between male and female patients

Male patients presented more back pain and leg pain compared to female patient (*p* = 0.001 and 0.002 separately), and there was no significant difference in ODI between male and female patients. (Table 1)

**Table 1:** Comparison of clinical presentation in patients with degenerative lumbar scoliosis of different genders

	Male	female	Statistics	*p*-Value
Cases	41	158		
Age (years)	66.1 ± 6.6	62.9 ± 6.3	2.850	0.005*
VAS-back pain	66.5 ± 6.4	53.1 ± 10.8	4.064	0.001*
VAS-leg pain	64.4 ± 10.9	54.0 ± 10.1	3.131	0.002*
ODI	66.7 ± 6.5	64.5 ± 6.2	1.097	0.274

*p < 0.05

### Comparison of coronal spinal parameters between male and female patients

Male patients presented smaller Cobb’s angle and less apex vertebrae rotation when compared to female patients (*p* = 0.003 and 0.011 separately), and there was no significant difference in scoliosis orientation, CBD, location of upper end vertebrae, location of lower end vertebrae, location of apex vertebrae, number of vertebrae included in the scoliosis, AVT between male and female patients. (Table 2)

**Table 2:** Comparison of coronal spinal parameters in patients with degenerative lumbar scoliosis of different genders

		Male	Female	Statistics	*p*-Value
Scoliosis orientation (cases)					
	Left	21	100		
	Right	20	58	1.99	0.158
Type of coronal imbalance (cases)					
	A	33	111		
	B	6	23		
	C	2	24	3.121	0.21
Upper end vertebrae (cases)					
	T11	1	3		
	T12	6	23		
	L1	17	97		
	L2	17	35	6.950	0.074
Lower end vertebrae (cases)					
	L3	2	26		
	L4	39	132	3.609	0.057
Number of vertebrae included in the scoliosis (cases)					
	3	17	54		
	4	17	85		
	5	7	19	2.085	0.352
Rotation of apex vertebrae (cases)					
	1	18	30		
	2	8	42		
	3	9	53		
	4	6	33	11.225	0.011*
Cobb’s angle		24.8 ± 9.8	30.1 ± 10.1	−3.012	0.003*
Coronal balance distance		17.7 ± 15.6	20.4 ± 14.3	−1.405	0.297
Apex vertebrae		2.5 ± 0.78	2.5 ± 0.70	−0.553	0.582
Apical vertebral translation		1.9 ± 1.0	2.2 ± 0.9	−1.759	0.080

*p < 0.05

### Comparison of sagittal spinal parameters between male and female patients

Male patients presented larger LL (*p* = 0.046) and smaller PI-LL (*p* = 0.022) when compared to female patients, and there was no significant difference in SVA, TL, PI, SS and aLL between male and female patients. (Table 3)

**Table 3:** Comparison of sagittal spinal parameters in patients with degenerative lumbar scoliosis of different genders

	Male	Female	Statistics	*p*-Value
Sagittal vertical axis (mm)	50.9 ± 38.2	49.9 ± 42.2	0.132	0.895
Lumbar lordosis (degrees)	33.3 ± 15.7	27.7 ± 15.5	2.012	0.046*
Thoracolumbar kyphosis (degrees)	18.7 ± 13.5	15.4 ± 14.8	1.289	0.199
Pelvic incidence (degrees)	49.4 ± 11.0	50.6 ± 13.6	−0.511	0.610
PI-LL (degrees)	16.2 ± 15.5	22.9 ± 16.7	−2.304	0.022*
Sacral slope (degrees)	29.0 ± 9.4	26.0 ± 11.1	1.578	0.116
Apex of lumbar lordotic	4.3 ± 0.69	4.2 ± 1.06	0.438	0.662

PI-LL, pelvic incidence minus lumbar lordosis. *p < 0.05

### Comparison of paraspinal muscles FIR between male and female patients

Female patients presented higher FIR both in concave and convex side at apical vertebrae. There was no significant difference in HU value at L1 vertebrae between male and female patients. (Table 4)

**Table 4:** Comparison of vertebral BMD and paraspinal muscle degeneration in patients with degenerative lumbar scoliosis of different genders

	Male	Female	Statistics	p-Value
HU at L1 vertebrae	134.0 ± 48.2	132.1 ± 76.9	0.063	0.95
FIR at apical vertebrae				
Concave	24.9 ± 8.4	43.4 ± 16.4	−3.288	0.002*
Convex	23.0 ± 10.7	40.3 ± 18.2	−2.761	0.007*

*p < 0.05

### Correlation among differential parameters in male and female patients

**Fig. 5:**
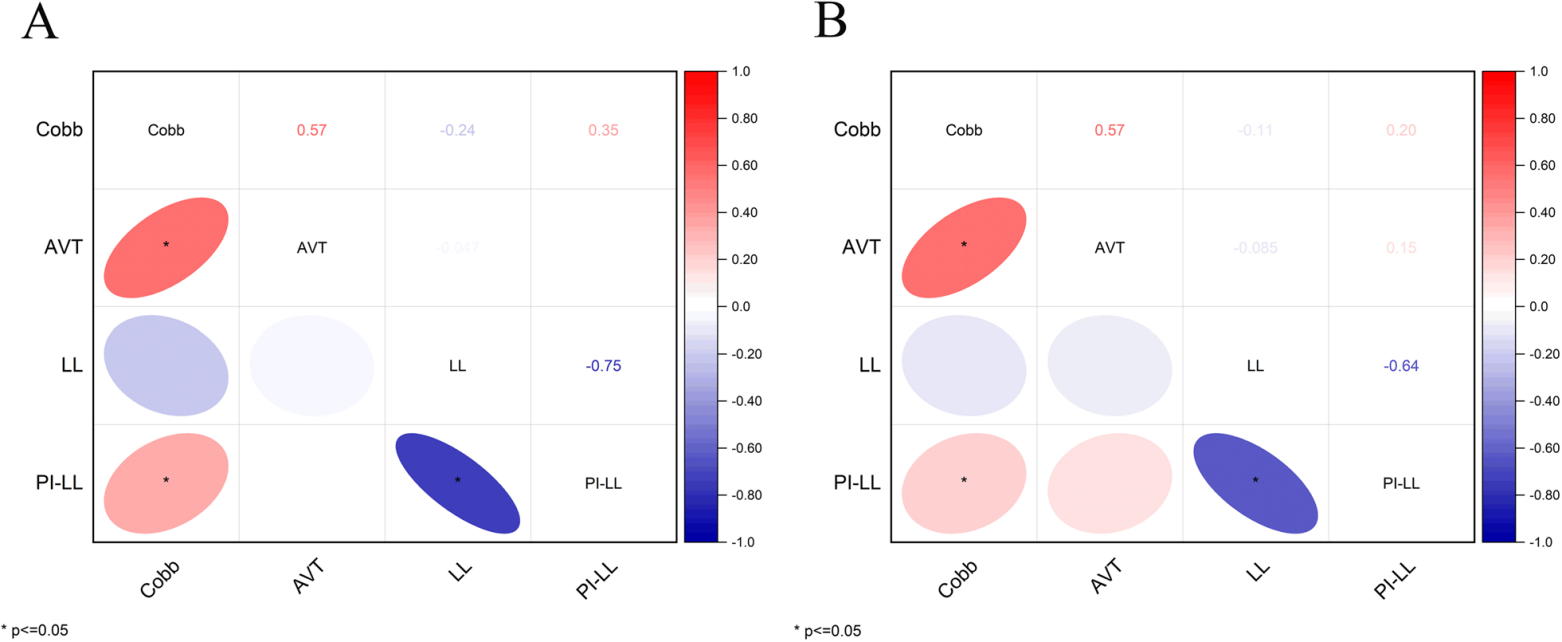
Heat map of correlations among different parameters. **A** Correlation among differential parameters in male patients. **B** Correlation among differential parameters in female patients

Both in the male and female patient groups, AVT and PI-LL were positively correlated with Cobb (*p* < 0.05), and PI-LL was negatively correlated with LL (*p* < 0.05) (Fig. 5)

## Discussion

In this cross-sectional study, male patients presented more back pain and leg pain compared to female patients in the clinical presentation and presented smaller Cobb’s angle, less apex vertebrae rotation, larger LL, smaller PI-LL and lower paravertebral muscle FIR when compared to female cohorts in radiological characteristics, and we speculate that the difference of these parameters between male and female patients may be of potential etiological significance for the DLS.

A large body of the literature clearly demonstrates that men and women respond differently to pain, with increased pain sensitivity and risk for clinical pain commonly being observed among women [10]. In the present study, male DLS patients, however, showed more severe low back pain and leg pain than female patients. Based on the findings of Nakamae et al [11]. that low back pain due to DLS is not directly related to the patient’s age or the severity of scoliosis, we believe that a study by Robinson and colleagues [12] gives a more plausible explanation. Since women are more sensitive to pain than men, men are more reluctant to report pain. Therefore, in the present cross-sectional study, unlike female patients, male patients with DLS opted for hospitalization only when they experienced more severe pain.

The natural progression of degenerative scoliosis with age is inevitable. A study of 51 patients with early degenerative lumbar scoliosis followed for 13.7 years found that the patients’ scoliosis curve progressed by an average of 0.4° to 1.4 °per year [13]. The mean curve progression over 5 years was reported to average 3 degrees per year in 73% of patients, Grade 3 apical rotation, a Cobb angle ≥30 degrees, lateral vertebral translation ≥6 mm and prominence of L5 in relation to the intercrest line were important factors for predicting curve progression [14]. In the current cross-sectional study, however, male DLS patients were relatively older than female patients but had a smaller Cobb angle compared to the female cohort. We propose that the effect of gender differences on scoliosis progression is much greater than the effect of natural progression. As a cross-sectional study, we strictly followed the inclusion and exclusion criteria to screen patients within 10 years of our institutional case base and still obtained results that male patients were on average older than female patients. One possible reason for this is that the prevalence of DLS is greater in female than in male patients, and the male sample size results in an unavoidable selection bias. However, another more plausible explanation is that the age of onset of DLS may be greater in male patients than in females. According to Xu et al.’s study [5], osteopenia, gender of female, and aged older than 65 years could contribute to the presence of DLS. In this study, the BMD (L1 vertebral HU value) of male subjects was not significantly different from female subjects and the mean value was smaller in female subjects. This interpretation is also supported by the fact that the Cobb angle was significantly smaller in male subjects than in female subjects. And another possible explanation for the larger Cobb angle in female subjects than male subjects is that gender differences in muscle atrophy play a role. One possible explanation is that gender differences in muscle atrophy play a role. Studies have shown that aging affects muscle function more in women than in men, and that skeletal muscle attachment quality is lower in older women compared to men [15]. It is generally accepted that the paraspinal muscles are the dynamic stabilizers of the spine column [16]. Research by both Crawford et al. [17] and Urrutia et al. [18] showed that fat infiltration of the paraspinal muscles was greater in females than in males in all age groups. A study by Yagi et al. [19] concluded that there is a causal relationship between paraspinal muscle degeneration and the development of DLS. In the present study, the paraspinal muscles of male patients were significantly less degenerated than those of female patients. We believe that this may be one of the reasons for the different severity of scoliosis in male and female DLS patients. The third possible reason is that the dramatic decrease in estrogen production in women after menopause also plays a role. It is now generally accepted that disk degeneration is the initiating factor in DLS [20]. Our team has long been engaged in research on the correlation between estrogen and intervertebral disk degeneration and found that estrogen has an excellent ability to inhibit the apoptosis of intervertebral disk cells and revealed the related mechanism of action [21, 22]. The female patients in this cross-sectional study were of postmenopausal age, and the rapid decline in estrogen secretion led to the loss of estrogen protection of the disk cells, which accelerated the degeneration of the disks and thus the progression of DLS, which may be another reason for the severity of scoliosis in the female patients compared with the male patients.

Different studies have reached varied conclusions regarding the correlation between apex vertebrae rotation and DLS progression. Pritchett et al. [14] and Kohno et al. [23] suggest that apex vertebrae rotation is a predictor of curve progression. In contrast, Park et al.’s research [13] found no significant correlation between apex vertebrae rotation and scoliotic angle in the early stages of DLS. In this study, we found a significant correlation between apical vertebrae rotation and coronal Cobb angle in both males and females. Combined with previous studies, we believe that the apex vertebrae rotation does not precede the appearance of scoliosis, but occurs with the progression of scoliosis.

Pelvic incidence (PI), first described by Duval Beaupere, is unique for skeletal mature individuals and is not affected by posture [24]. Since PI is the sum of sacral slope (SS) and pelvic tilt (PT), it is a valid descriptor of the global shape of the pelvis and the position of the sacrum in the pelvic unit [25]. Pelvic incidence minus lumbar lordosis (PI-LL) was used to quantify the mismatch between pelvic morphology and lumbar curve [26, 27]. In this study, there was no difference in PI values between male and female DLS patients, LL was greater in male patients than in female patients, and PI-LL was less in male patients than in female patients according to geometric calculations. In addition, the PI-LL values of male and female patients were positively correlated with their Cobb angles. Han et al. [28] found significantly lower LL values in DLS patients compared to normal subjects in a comparative study. A prospective study by Jimbo et al. [29] concluded that the decrease in LL is a result of the progression of lumbar degeneration and leads to further deformities. These studies all support our conclusion that it is precisely because female patients exhibit a more severe scoliosis angle than male patients, which in turn results in smaller LL values, and larger PI-LL values. Furthermore, the PI-LL mismatch was higher in female than male, and ODI showed no statistical difference according to sex. This findings suggested that sex-based difference can impact manifestation of DLS. It also strengthens our conclusion that low back pain was more pronounced in male patients and scoliosis was more severe in female patients.

In previous research on the pathogenesis of DLS patients, male and female patients were usually analyzed as a whole. Our current research shows that the clinical manifestations of male and female DLS patients are not identical. We believe that the influence of gender should be considered in future research on the pathogenesis of DLS patients.

We recognize that the current study still has limitations. As it was a retrospective study, we could not provide strong evidence of causality for the various differences between male and female DLS patients. However, as a cross-sectional study, we can still demonstrate that gender factors play a role in the development of scoliosis in DLS. In addition, our study was a single-center study, and the patients were all from the north of China, and whether the findings can be generalized to other ethnic groups still needs to be confirmed by further multi-center large-sample studies.

## Conclusion

In summary, gender differences do exist in DLS patients with regard to clinical and radiological presentation, low back pain was more pronounced in male patients and scoliosis was more severe in female patients based on this cross-sectional study.

## Data Availability

The datasets analyzed during the current study are available from the corresponding author on reasonable request.

## Abbreviations

DLS: Degenerative lumbar scoliosis
P/A: Postero-anterior
HU: Hounsfield unit
BMD: Bone mineral density
VAS: Visual analog scale
ODI: Oswestry Disability Index
AV: Apex vertebrae
AVT: Apical vertebral translation
CBD: Coronal balance distance
CSVL: Central sacral vertical line
SVA: Sagittal vertical axis
TLK: Thoracolumbar kyphosis
LL: Lumbar lordosis
PI: Pelvic incidence
SS: Sacral slope
aLL: Apex of lumbar lordotic
FIR: Fatty infiltration rate
